# Rheopectic Behavior for Aqueous Solutions of Megamolecular Polysaccharide Sacran

**DOI:** 10.3390/biom10010155

**Published:** 2020-01-17

**Authors:** Fitri Adila Amat Yusof, Miho Yamaki, Mika Kawai, Maiko K. Okajima, Tatsuo Kaneko, Tetsu Mitsumata

**Affiliations:** 1 Graduate School of Science and Technology, Niigata University, Niigata 950-2181, Japan; fitriadilaamat@gmail.com (F.A.A.Y.); giallo_80kin@ezweb.ne.jp (M.Y.); mikagoro@eng.niigata-u.ac.jp (M.K.); 2 Graduate School of Advanced Science and Technology, JAIST, Nomi 923-1292, Japan; k-maiko@jaist.ac.jp (M.K.O.); kaneko@jaist.ac.jp (T.K.)

**Keywords:** polysaccharide, polyelectrolyte, viscosity, rheopexy, thixotropy, negative thixotropy

## Abstract

The rheopectic behavior of sacran aqueous solutions, a natural giant molecular polysaccharide with a molecular weight of 1.6 × 10^7^ g/mol, was investigated. When a low shear was applied to 1.0 wt.% sacran solution, the shear viscosity increased from 7.2 to 34 Pa·s. The increment in the viscosity was enhanced as the shear rate decreased. The shear viscosity was independent of the time at a shear rate of 0.8 s^−1^; simultaneously, thixotropic behavior was observed at shear rates higher than 1.0 s^−1^. A crossover was observed at 0.15 wt.% for the concentration dependence of both the viscosity increase and zeta potential, which was the vicinity of the helix transition concentration or gelation concentration. It was clear that the molecular mechanism for the rheopexy was different at lower and higher regions of the crossover concentration.

## 1. Introduction

Sacran is a supergiant cyanobacterial polysaccharide extracted from the jelly extracellular matrix of a river plant named *Aphanothece sacrum* [1], which has been mass cultivated in Japan for a long time. Sacran is known for its extremely high absolute molecular weight of 1.–2.2 × 10^7^ g/mol [2,3], with a chain length more than 30 μm [4]. The saccharide chain contains carboxylate groups (17 mol%) and sulfate groups (12 mol%) relative to the sugar residues [2]. That is, the aqueous solution of sacran is a polyelectrolyte. Due to the electric charges on the chain, sacran changes its conformation depending on the sacran concentration, e.g., helix transition concentration, where the sacran chain change from random coil to double helix occurs at 0.09 wt.% and gelation concentration, where sacran showed a transition from liquid to weak gel at 0.25 wt.%.

Sacran shows various unique phenomena such as high swelling [2,4,5] and anisotropic swelling behaviors [4], liquid crystalline (LC) behavior [6,7], ion adsorption behavior [8,9,10], and anisotropic diffusion behavior in highly ordered sacran films [3,11,12,13,14]. The retention capacity for uncross-linked sacran exceeds higher than 6000 mL/g for pure water and 2700 mL/g for NaCl aqueous solution [2]. Similar high swelling behaviors were found for cross-linked sacran hydrogels, e.g., 6100 mL/g for pure water and 530 mL/g for 0.9 wt.% for saline solution [2]. These high-swelling behaviors might originate from the large mesh consisting of extremely long chains of sacran or macroscopic LC domains with millimeter to several centimeter scales in which sacran chains are aligned by self-organization [6]. For ion adsorption, sacran shows a feature that the adsorption ratio for sacran against trivalent neodymium ions (Nd^3+^) is twice that for sodium alginate [9]. The efficient adsorption for lanthanoid ions was developed to a hydrogel consisting of sacran and polyvinyl alcohol (PVA) [10]. Interestingly, sacran gels cross-linked by trivalent metal ions demonstrate photo shrinkage behavior by irradiating ultraviolet light [8]. There are some interesting reports describing the bio-inspired texture for dried sacran films obtained by evaporating water from their aqueous solution [11,12,13,14]. Thus, sacran demonstrates various unique phenomena originating from the feature of super megamolecules. Anomalous property can also be seen in the rheological properties of sacran; that is, the viscosity increased over time when a steady shear with low shear rates was applied.

Many reports describe the phenomenon of negative thixotropy or rheopexy, which is the increase of viscosity with time under a fixed-shear rate. Our understanding about these phenomena is the negative thixotropy is a phenomenon where the absolute value of viscosity decreased after switching from a high shear rate to a low shear one. Meanwhile, the rheopexy is a phenomenon that the absolute value of viscosity increases after the switching. With both negative thixotropy and rheopexy, the viscosity increases over time. A typical example of the negative thixotropy is found in a paper for ferric oxide reported by Kanai et al. [15]. There are many reports dealing with “negative thixotropy”. For example, polyacrylamide [16,17,18,19] and poly(methyl methacrylate) [20] demonstrate negative thixotropy at a shear rate of 10^−3^ s^−1^. A potato starch paste exhibits the negative thixotropy at a relatively high shear rate (50, 300 s^−1^) and high temperature (80–121 °C) [21,22]. The aqueous solution of polymers containing inorganic particles demonstrates the negative thixotropy depending on the rest time between the pre-shear and viscosity measurements [23] or the value of pH [24,25]. On the other hand, a paper for highly concentrated emulsion by Zhao et al. is a typical example of rheopexy [26]. There are some reports dealing with “rheopexy,” e.g., polyacrylamide with high molecular weight [27]. A tomato juice containing 1% soy protein demonstrates a rheopectic behavior at shear rates lower than 250 s^−1^ due to an enhanced aggregation between pectins and soy protein [28]. A synovial fluid and bovine serum albumin (BSA) solution demonstrates rheopectic behavior at shear rates below 10^−1^ s^−1^ due to protein aggregation [29]. These papers tell us that rheological behavior is complex and many factors affect this phenomenon, e.g., associations or aggregation of macromolecules by their deformation and orientation due to the shear. However, the origin is not yet well understood.

In this study, we investigated the rheopectic behavior for sacran aqueous solutions at various shear rates and various sacran concentrations. The sacran concentration dependence of the zeta potential was also studied. We discuss here the relationship between the viscosity increase and physicochemical properties such as dielectric property or chain conformation. The influence of the macroscopic LC domain on the rheopectic behavior is also discussed.

## 2. Experimental Procedures

### 2.1. Sacran Extraction and Solution Preparation

Sugar chains were extracted from *Aphanothece sacrum* via a previously reported method [1,2] explained as follows. The *Aphanothece sacrum* samples were freeze–thawed and washed in pure water, followed by lyophilization. The samples were washed three times using a large amount of ethanol with shaking (120 rpm) overnight, and then collected by filtration using gauze. The ethanol-washed samples were put into 0.1 M NaOH aq. at 100 °C and agitated at constant temperature for 4 h to yield the transparent solution. The solution was dialyzed with pure water for more than 72 h using the regenerated cellulose membrane (molecular weight cut-off (MWCO): 14,000) until the pH value decreased to 8.0–9.0 and then filtrated. Then, the filtrate was concentrated by a rotary evaporator to create a highly viscous solution. The viscous solution was slowly poured into 100% isopropanol (1000 mL) to precipitate a white fibrous material. The fibers were dissolved in hot water again, concentrated, and reprecipitated. These operations were repeated three times in total. The fibrous precipitates in isopropanol were collected and dried using a vacuum oven. The dried sacran was completely dissolved in pure water at 100 °C and cooled at room temperature to obtain a sacran aqueous solution of 1 wt.%. It was diluted to prepare a sacran solution with various concentrations. Solutions stored in the refrigerator longer than 24 h were heated again before taking the rheological measurements.

### 2.2. Rheological Measurements

#### 2.2.1. Steady-Shear Viscosity Measurement

Viscoelastic measurements were carried out using a rheometer (MCR 301, Anton Paar) with a stainless cone plate, which had a diameter of 50 mm (CP50, Anton Paar). The minimum torque that can be detected by the rheometer is 0.1 μN·m. The temperature was controlled at 25.0 ± 0.1 °C using a Peltier plate during the viscoelastic measurement. Steady-shear flow viscosity experiments were performed at a range of shear rate from 0.001 to 10 s^−1^. A pre-shear with a shear rate of 100 s^−1^ was applied for 10 s then let rest for 180 s before the measurement in order to reset the structure of self-organized LC domains. The measurement was performed for 900 s until an equilibrium of apparent viscosity was achieved.

#### 2.2.2. Dynamic Viscoelastic Measurement

Dynamic viscoelastic measurements were also carried out using the same rheometer with a shear of 0.01 at 0.1 Hz. The strain dependence of the storage modulus revealed that the 1% sacran aqueous solution showed the linear viscoelastic regime up to a strain of 0.2. The temperature was controlled at 25.0 ± 0.1 °C. Storage modulus was also measured in this experiment at three points: before shear (before steady shear was applied), immediately after the shear (at 900 s), and the end of the measurement (at 1726 s). The frequency sweep test of dynamic modulus for 1 wt% sacran aqueous solution revealed that the storage modulus G’ was higher than the loss modulus G” in a frequency range of 0.01–10 Hz.

### 2.3. Zeta Potential Measurement

The zeta potential was measured by a micro-electrophoresis zeta potential analyzer (Model 502, Japan Rufuto Co., Ltd.) at room temperature (20–23 °C). An aqueous sacran solution with 5 mL was poured into a cell with an electrode distance of 4.88 cm, and an electric voltage of 20 V was applied to the electrodes. Image analysis was carried out using software (Move-tr/2D, Library Co. Ltd.) to analyze the movement of polysaccharides. The zeta potential was calculated from ζ = *η*μ/ε_0_ε_r_, where the *η*, μ, ε_0_, and ε_r_ are viscosity, mobility, dielectric constant of solution, and dielectric constant of vacuum, respectively.

### 2.4. Observation of LC Domains

A 1 mm thick silicon sheet with a hole of φ25 mm was placed on a glass plate, and the aqueous solution of 1 wt.% sacran was poured in the hole and sandwiched by a glass plate. Two polarized films were arranged so as to be a condition of crossed nicols, and the polarized image was recorded by a digital camera.

## 3. Results and Discussion

### 3.1. Rheopectic Behavior

**Figure 1:**
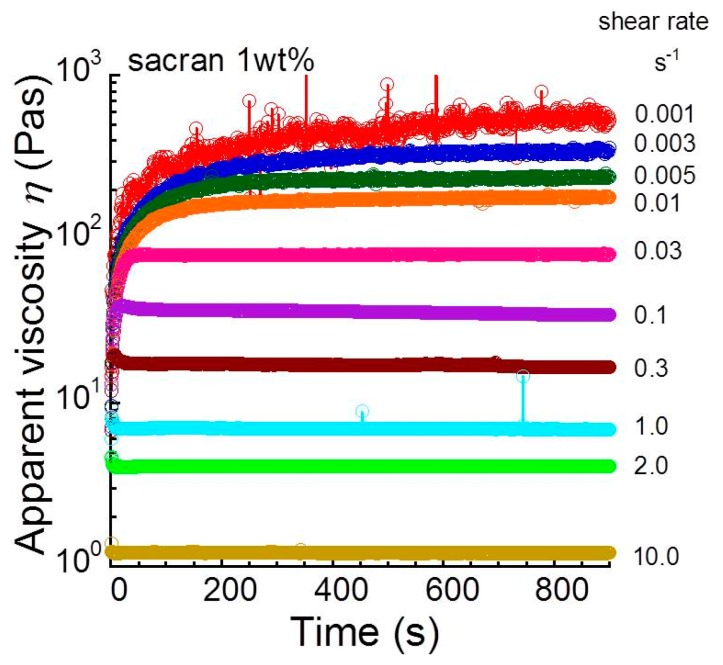
Time profiles of apparent shear viscosity for 1 wt.% sacran aqueous solution as a function of shear rates.

Figure 1 shows the time profiles of apparent shear viscosity for 1 wt.% sacran aqueous solution as a function of shear rates. A phenomenon called rheopexy was observed at low shear rates, where the shear viscosity increased with an elapse of time, and it became constant within 900 s. Similar behavior was reported in, e.g., polyacrylamide [27], with the increase in the viscosity of ~6 Pa·s. However, the viscosity increase for the sacran solution was extremely huge. The increment in the viscosity was enhanced as the shear rate decreased. The shear viscosity was found to be independent of the time at a shear rate of 0.8 s^−1^. Meanwhile, at shear rates higher than 1.0 s^−1^, the shear viscosity rapidly decreased in the first 10 s, which is called (positive) thixotropy. The sacran chain aligned in the flow direction at both low and high shear rates. Therefore, the behavior of viscosity changes is strongly influenced by the time constant of bonding between sacran chains, meaning the viscosity increase occurs only when the shear rate is enough to be shorter than the time constant of bonding.

**Figure 2:**
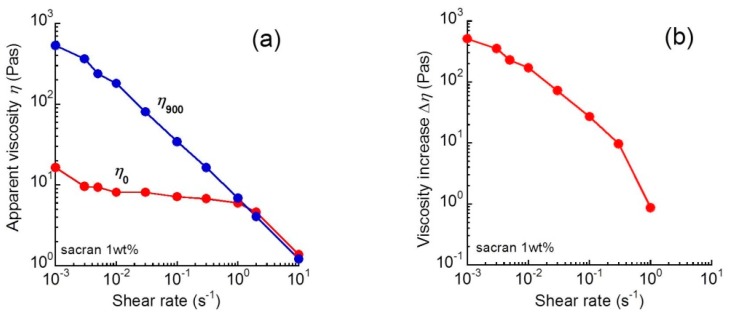
(**a**) Relationship between shear rate and apparent shear viscosity at 0 and 900 s; (**b**) relationship between the viscosity increase and shear rate for 1 wt.% sacran aqueous solutions.

Figure 2a demonstrates the relationship between shear rate and the apparent shear viscosity at 0 and 900 s for 1 wt.% sacran aqueous solution. The shear viscosity at 0 s, *η*_0_, for the sacran solution was almost independent of the shear rate at $\dot{\gamma }$ < 1, and it decreased remarkably with the shear rate at $\dot{\gamma }$ > 1. This indicates that the chains are extended immediately after applying the shear at $\dot{\gamma }$ > 1. Meanwhile, the shear viscosity at 900 s, *η*_900_, decreased monotonously with the shear rate, indicating that the sacran chains are fully extended in the flow direction. It is also clear from this figure that the rheopectic behavior does not occur at $\dot{\gamma }$ > 1.

Figure 2b exhibits the relationship between the viscosity increase and shear rate for 1.0 wt.% sacran aqueous solution. The viscosity increase ∆*η* was defined by the following equation:

∆*η* = *η*_900_ − *η*_0_ (1)

where *η*_0_ and *η*_900_ are the shear viscosity at 0 and 900 s, respectively. The viscosity increase for sacran solutions decreased significantly with the shear rates.

**Figure 3:**
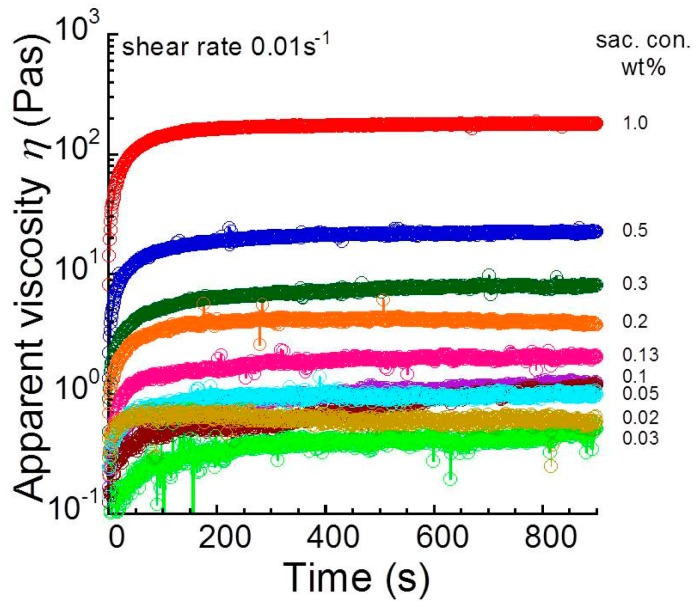
Time profiles of apparent shear viscosity for sacran aqueous solutions with various sacran concentrations at a shear rate of 0.01 s^−1^.

Figure 3 indicates the time profiles of apparent shear viscosity at a shear rate of 0.01 s^−1^ for sacran aqueous solutions with various sacran concentrations. Similar to Figure 1, the shear viscosity increased with an elapse of time, and it became constant within 900 s. The increment in the viscosity was enhanced as the concentration increased. The minimum sacran concentration demonstrating the rheopectic behavior was found to be approximately 0.02 wt.%.

**Figure 4:**
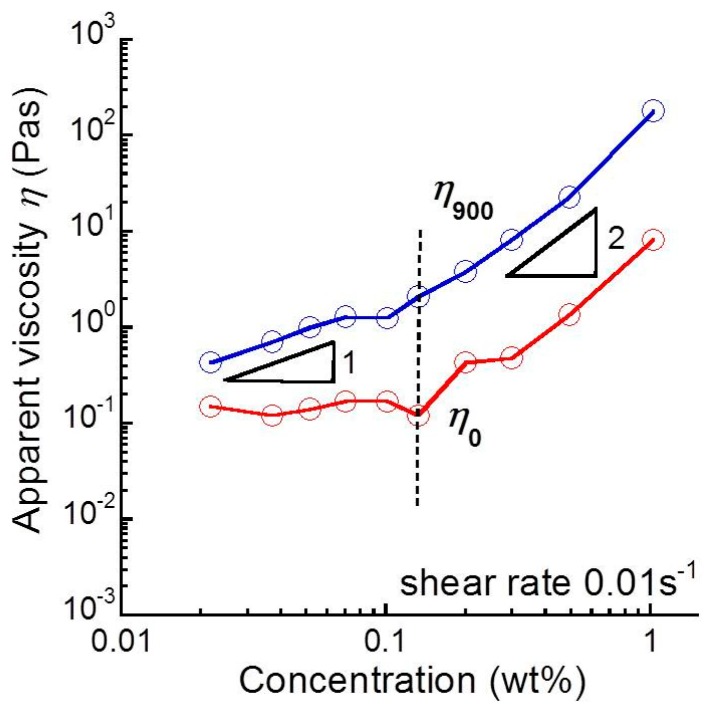
Relationship between sacran concentration and apparent shear viscosity at 0 and 900 s (shear rate: 0.01 s^−1^).

Figure 4 shows the relationship between sacran concentration and the apparent shear viscosity at 0 and 900 s. The shear viscosity at 0 s, *η*_0_, was almost independent of the concentration at *c* < 0.15 wt.%. Meanwhile, it increased remarkably as ~*c*^1.9^ at c > 0.15 wt.%. At 900 s, the shear viscosity, *η*_900_, increased, with the concentration showing the concentration dependence as ~*c*^0.9^ at c < 0.15 wt.% and ~*c*^2.2^ at *c* > 0.15 wt.%. 

### 3.2. Recovery from Aligned State

**Figure 5:**
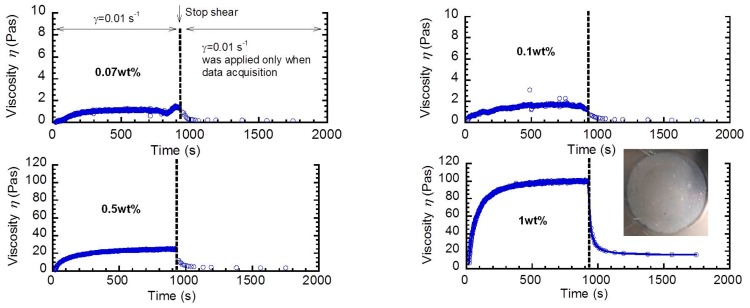
Recovery in the shear viscosity for sacran aqueous solutions with various sacran concentrations. Inset: Polarized photo for 1 wt.% sacran aqueous solution taken under the cross nicol.

Figure 5 presents the recovery in the apparent shear viscosity for sacran aqueous solutions with various concentrations. A shear with a shear rate of 0.01 s^−1^ was applied in the first 900 s, and then the steady-shear was stopped at 900 s. Then, the shear viscosity was measured only when the data were acquired by applying the steady-shear for 5 s. As noted in Figure 3, the shear viscosity increased with an elapse of time and it became constant within 900 s. After stopping the shear at 900 s, the shear viscosity decreased with time, almost recovering to a value that was slightly higher than the initial value. This strongly suggests that the aligned state is not thermodynamically stable, and the bonding energy between sacran chains is not strong compared to the thermal energy *k*_B_*T*. The inset of Figure 5 indicates a polarized photo for 1 wt.% sacran aqueous solution taken under the cross nicol using two sheets of polarized films. There was no white region observed for the solution indicating the macroscopic LC domains of aligned chains of sacran. Therefore, it can be considered that there is no direct relationship between macroscopic LC domain and rheopectic behavior or the recovery in the viscosity from the aligned state.

**Figure 6:**
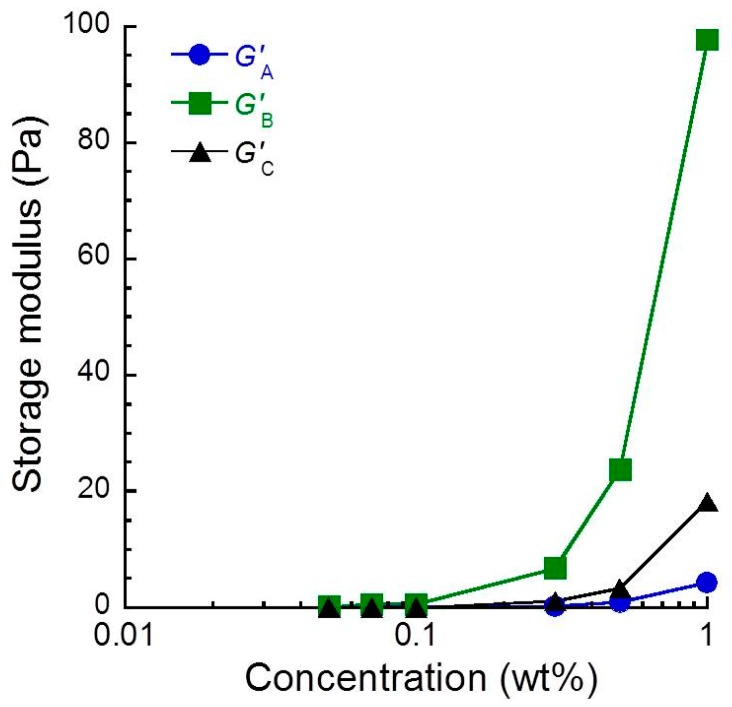
Effect of the low shear on the storage modulus for sacran aqueous solutions as a function of sacran concentrations.

The storage modulus for sacran solution was also measured in this experiment at three points: before the shear *G’*_A_, immediately after the shear *G’*_B_, and the end of experiment *G’*_C_ (data are shown in Table 1). The storage modulus *G’* can be explained by the following equation:

*G*’ = *υk*_B_*T* (2)

where *υ*, *k*_B_, and *T* are the number of cross-linking points, Boltzmann constant, and absolute temperature, respectively. Figure 6 exhibits the effect of low shear on the storage modulus for sacran aqueous solutions as a function of sacran concentrations. It was observed for all solutions that the *G’*_B_ was higher than *G’*_A_. This suggests that the cross-linking between sacran chains occurs due to the low shear. Therefore, it can be considered that the rheopectic behavior was caused by the cross-linking (physical bonding) between sacran chains aligned by the low shear. It was also observed for aqueous solutions higher than 0.3 wt.% that the *G’*_C_ was higher than *G’*_A_. This suggests that the cross-linking between sacran chains remained even after the shear was stopped. It is worth mentioning that the *G’*_B_ was slightly higher than *G’*_A_, even though the sacran concentration was below 0.1 wt.% (see Table 1), suggesting that only a few sacran chains make cross-linking with each other. It is clear for all solutions that the *G’*_C_ was lower than *G’*_B_, indicating that the physical bonds produced by the low shear were transient. Both the values of *G’*_A_ and *G’*_B_ were almost independent of the sacran concentration at *c* < 0.1 wt.%, suggesting that the number of bonds was constant for these solutions. 

**Figure 7:**
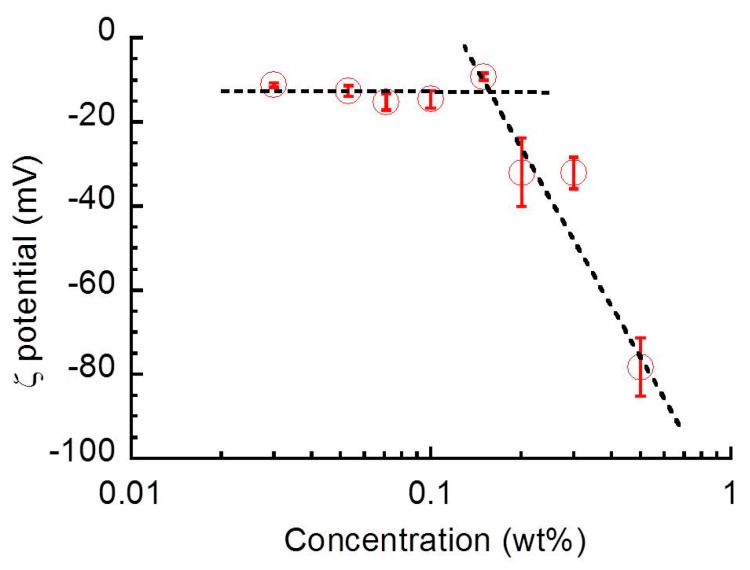
Sacran concentration dependence of the zeta potential for sacran aqueous solutions with various concentrations.

Figure 7 demonstrates the concentration dependence of zeta potential for sacran aqueous solutions with various concentrations. The value of η_0_ in Figure 2a was used for calculating the zeta potential. The zeta potential showed a crossover at 0.15 wt.%––it was constant at *c* < 0.15 wt.%, and it significantly increased with the concentration at *c* > 0.15 wt.%. In our previous study [6], the characteristic concentrations for sacran aqueous solution such as helix transition or gelation concentration are seen in a concentration range of 0.1 < *c* < 0.2 wt.%. We explained in the previous papers [6,7] that the sacran chains lost their electric charges at these concentrations and demonstrated structural changes such as helix transformation or gelation. The result of zeta potential obtained here may contradict this idea because the strong repulsion between the chains hinders the formation of helix or gel. However, it would be natural that the electric charges on the surface of the particle measured in the zeta potential experiment are different from the microscopic electric charges of the sacran chain obtained by dielectric spectroscopy. In any case, the mechanism for the structural changes of sacran seen at the crossover from semi-dilute regime to condensed regime (weak gel) must be reconsidered.

**Figure 8:**
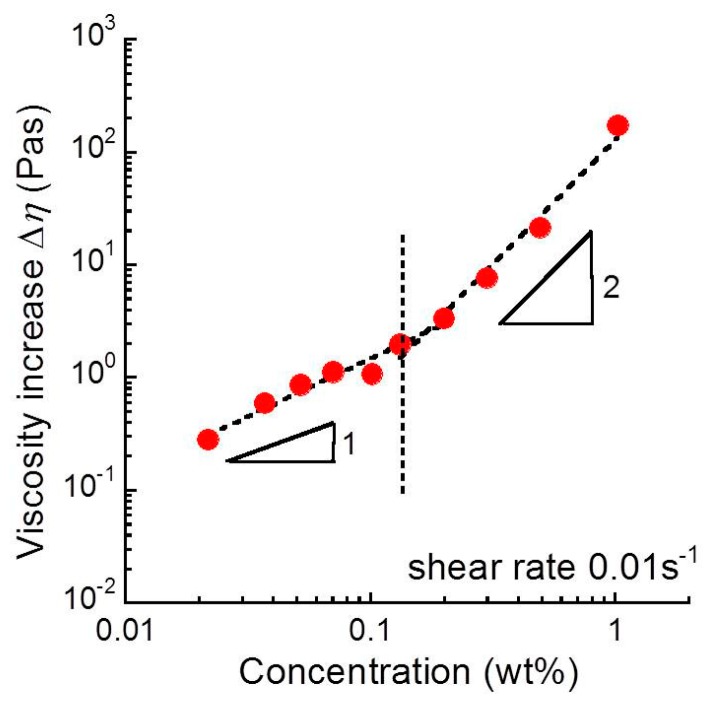
Relationship between the viscosity increase and sacran concentration for sacran aqueous solution.

Figure 8 represents the relationship between the viscosity increase and sacran concentrations for sacran aqueous solutions. The viscosity increase for sacran solutions ∆*η* was calculated from Equation (1). The increase in the viscosity increased with the concentration as ~*c*^1.0^ at *c* < 0.15 wt.% and as ~*c*^2.2^ at *c* > 0.15 wt.%. The crossover concentration of the viscosity increase was 0.15 wt.%, which equals the helix transition concentration seen in the zeta potential. This indicates there should be two different mechanisms for the rheopectic behavior relating to the conformation of sacran chains. As described in Figure 6, the viscosity increase originates from the transient cross-linking between sacran chains. At *c* < 0.15 wt.%, the viscosity increase was proportional to the sacran concentration (∆*η*~c^1.0^), suggesting that the number of cross-linking is proportional to the concentration. Accordingly, it is considered that the number of cross-linking increases with the sacran concentration (*N*_b_∝c), although the dependency of *G’* vs. *c* is very weak. At *c* > 0.15 wt.%, the viscosity increase was proportional to the square of sacran concentration (∆*η*~*c*^2.2^). This strongly indicates that the number of cross-linking points varies depending on the sacran concentration. To explain the square dependence of the viscosity increase, we considered a hypothesis that the number of bonding cites between chains increases in proportion to the sacran concentration (*N*_b_∝c). Therefore, it can be considered that the viscosity increase is caused by the cross-linking between the bonding cites, which were produced by the helix formation. The bonding cites might be the hydrophobic part in the sacran chain; however, the mechanism is not clear now. 

**Figure 9:**
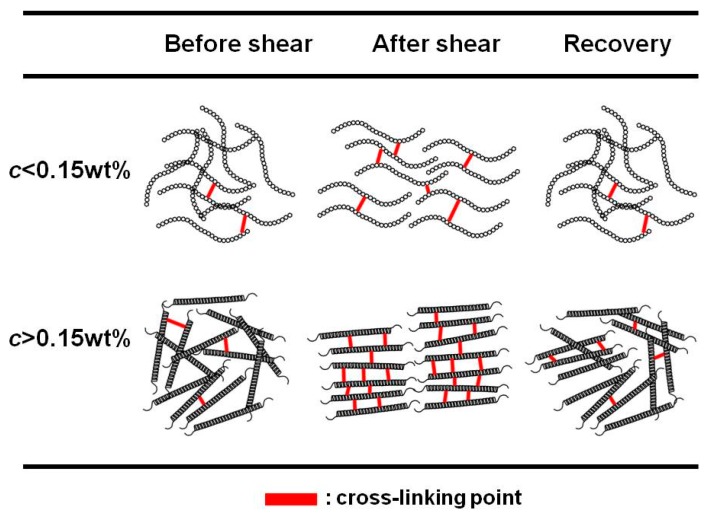
Schematic illustrations representing the rheopectic behavior for sacran aqueous solutions.

Figure 9 shows the schematic illustration for the rheopectic behavior for sacran aqueous solutions. As mentioned above, the rheopectic behavior was enhanced at 0.15 wt.%. The sacran chain demonstrated a transition concentration from random coil to double helix at approximately 0.1 wt.% and gelation concentration at 0.2 wt.% [6,7]. The zeta potential drastically decreased at the crossover concentration of 0.15 wt.%. It is quite natural to consider that the rheopectic behavior relates to the structural changes and macroscopic electric charges on the bundles of sacran chains. It might be that the rheopexy is mainly affected by the microscopic electric charges originating from counterion polarization. Otherwise, the hydrophobic interaction between sacran chains may be dominant in this system. It was clear that the molecular mechanism for the rheopexy is different at lower and higher regions of the crossover concentration. At concentrations below 0.15 wt.%, sacran chains before applying the shear are randomly distributed and there are few cross-linking points. When a low shear was applied, the sacran chains aligned in the flow direction and made a few cross-linking points between chains, resulting in the increase in the shear viscosity. After the shear was stopped, a clear change in the storage modulus was not observed compared to the initial state; therefore, the chains recovered to the random state again and cross-linking points disappeared. At concentrations above 0.15 wt.%, sacran chains before applying the shear were randomly distributed, and there were few cross-linking points. When a low shear was applied, the sacran chains aligned in the flow direction and made cross-linking points between the chains, resulting in the increase in the shear viscosity. After the shear was stopped, aligned sacran chains became random again, and most of the cross-linking points disappeared since the cross-linking was not strong enough to keep the aligned state. When a shear with high shear rate was applied, the sacran chains aligned in the flow direction. However, they could not make cross-linking points, resulting in the decrease in the shear viscosity (thixotropic behavior).

## 4. Conclusions

The rheopectic behavior for sacran aqueous solutions has been investigated via steady shear viscosity, dynamic viscoelastic, and zeta potential measurements. The shear viscosity for sacran aqueous solutions increased with an elapse of time at low shear rates. Meanwhile, it almost recovered to an initial viscosity after the shear stopped. It was also observed for these solutions that the storage modulus increased after the shear was applied. These results strongly indicate that the rheopectic behavior originated from weak and transient cross-linking between sacran chains. The concentration dependence of the changes in shear viscosity, storage modulus, and zeta potential showed a crossover concentration at 0.15 wt.%, suggesting that the mechanism of the rheopexy changed before and after the crossover concentration. Above the crossover concentration, the number of cross-linking points per unit chain may increase in proportion to the sacran concentration. Macroscopic LC domain was not observed for these aqueous solutions; therefore, it is clear that there is less relation between rheopectic behavior and macroscopic LC domain. We now investigate the attractive interaction or gelation mechanism for highly charged polysaccharide chains to elucidate the molecular mechanism for the rheopectic behavior.

## Figures and Tables

**Table 1:** Storage modulus for sacran aqueous solution with various concentrations: *G’*_A_: before shear; *G’*_B_: after shear; *G’*_C_: after recovery.

Sacran Concentration (wt.%)	Storage Modulus *G’* (Pa)		
	*G’*_A_	*G’*_B_	*G’*_C_
0.05	8.6 × 10^−2^	1.1 × 10^−1^	2.4 × 10^−2^
0.07	1. 3 × 10^−1^	6.7 × 10^−1^	9.4 × 10^−2^
0.1	1.2 × 10^−1^	5.8 × 10^−1^	2.9 × 10^−2^
0.3	2.2 × 10^−1^	6.7	1.1
0.5	9.6 × 10^−1^	24	3.2
1.0	4.4	92	18
